# Robust optimization of catheter positions and dwell times for HDR prostate brachytherapy

**DOI:** 10.1002/mp.70640

**Published:** 2026-08-18

**Authors:** Joseph B. Schulz, Bryan P. Bednarz, John M. Floberg, Jordan M. Slagowski

**Affiliations:** ^1^ Department of Medical Physics University of Wisconsin‐Madison Madison Wisconsin USA; ^2^ Department of Radiation Medicine University of Wisconsin‐Madison Madison Wisconsin USA

**Keywords:** brachytherapy, catheter placement, inverse planning, robust optimization

## Abstract

**Background:**

HDR prostate brachytherapy plans are sensitive to catheter placement uncertainty, as discrepancies between planned and delivered catheter positions can degrade target coverage and increase organ at risk dose. Current inverse planning methods optimize dwell times for a fixed implant geometry and do not account for geometric uncertainty during planning. Existing robust optimization approaches for HDR brachytherapy are similarly limited to dwell time optimization, and no framework has jointly optimized catheter configurations and dwell times while explicitly incorporating catheter insertion uncertainty.

**Purpose:**

To develop an analytical inverse planning method for prostate HDR brachytherapy that improves robustness to catheter placement uncertainty while maintaining nominal target coverage and organ at risk constraints.

**Methods:**

Overall, 32 previously treated prostate HDR cases were retrospectively analyzed. Dose was modeled with a TG‐43U1 influence matrix. A candidate catheter set was generated by adding four translated trajectories per clinical catheter at ±5mm in the anterior posterior and lateral directions, with dwell positions cropped to remain within the loading structure. Dwell times and catheter selection were optimized using a dose fidelity term with a group minimax concave penalty (gMCP) to enforce catheter level sparsity, and a surface restricted dose gradient penalty (SDGP) applied to organ at risk surface voxels. Four strategies were compared: clinical planning, dose fidelity+SDGP, dose fidelity+gMCP, and dose fidelity+gMCP+SDGP. Hyperparameters remained fixed for all patients, respectively. All nominal plans were normalized to PTV $V_{100\%}=95\%$. Robustness was evaluated using 1000 Monte Carlo trials per patient per uncertainty level with independent Gaussian catheter displacements in x,y,z and σ∈{0,0.5,1.0,1.5,2.0,2.5,3.0}mm. Endpoints included PTV $V_{100\%}$, bladder and rectum $V_{75\%}$, urethra $V_{125\%}$, and the clinical acceptability rate of the intersection of these metrics, using NRG‐GU009 HDR boost criteria.

**Results:**

Overall, the combined approach was comparable or an improvement to clinical positions alone. At σ=1.5mm, the complete approach with dose fidelity+gMCP+SDGP improved perturbed target coverage, with mean PTV $V_{100\%}=92.0\%\pm 1.1\%$ versus 89.5%±1.2% for clinical planning and 89.8%±0.9% for dose fidelity+gMCP. The corresponding acceptability rate was 77.7% for dose fidelity+gMCP+SDGP versus 50.0% for clinical planning.

**Conclusion:**

Coupling gMCP based catheter selection with an organ surface dose gradient penalty improved robustness of prostate HDR brachytherapy plans to independent catheter placement uncertainty, with comparable nominal plan quality after normalization.

## 1 INTRODUCTION

High‐dose‐rate brachytherapy (HDRBT) is a standard of care treatment option for localized prostate cancer, utilized either as monotherapy or as a boost to external beam radiotherapy (EBRT).^1^, ^2^, ^3^ Consensus guidelines promote HDR brachytherapy boost in combination with EBRT for intermediate‐ and high‐risk prostate cancer due to superior biochemical control relative to EBRT alone, and reduced side effects compared to low‐dose‐rate BT.^4^ HDRBT enables dose escalation to the planning target volume (PTV) with rapid dose fall off, potentially sparing the urethra, bladder, and rectum more effectively than EBRT alone.^2^ However, the steep dose gradients required for organ‐at‐risk (OAR) sparing simultaneously render the dose distribution highly sensitive to geometric uncertainties and implant motion.^5^, ^6^ Excessive dose to the urethra correlates with genitourinary toxicities, including stricture and urinary obstruction.^7^, ^8^

A primary source of uncertainty in prostate HDRBT is the discrepancy between planned and delivered catheter positions. Catheter insertion is affected by tissue deformation, needle deflection, and setup errors, and catheter migration between imaging and treatment delivery, or between fractions, can substantially degrade target coverage and increase OAR dose if not adequately accounted for.^6^, ^9^, ^10^, ^11^, ^12^ In current clinical practice, catheter placement and treatment planning are performed as independent steps. Catheters are first placed by the physician, after which inverse planning algorithms such as Inverse Planning by Simulated Annealing (IPSA) or Hybrid Inverse Planning and Optimization (HIPO) are used to optimize source dwell times for the given implant.^13^, ^14^, ^15^, ^16^ While current techniques produce clinically acceptable nominal plans, they do not consider catheter placement during optimization and do not explicitly account for geometric uncertainties in catheter position or intrafraction motion.^15^, ^17^ These limitations motivate strategies that explictly integrate catheter placement decisions with dwell time optimization to improve treatment plan quality.

Several studies have investigated algorithmic approaches for simultaneous catheter position selection and dwell time optimization in HDRBT. Gorissen et al. formulated mixed integer models that optimize catheter positions and dwell times with DVH based criteria.^18^ Wang et al.^19^ introduced a group sparsity framework using $L_{2,1}$ norm regularization to jointly select needles and optimize dwell times. Frank et al.^20^ further developed analytical catheter selection to improve selection behavior and convergence. Separately, recent work has highlighted clinically relevant catheter curvature and nonlinearity in prostate HDR implants, motivating planning models that move beyond rigid straight catheter assumptions.^21^, ^22^

To address geometric and anatomical uncertainties, robust optimization techniques originally developed for EBRT have been adapted to HDRBT. Balvert et al.^23^ formulated a worst case robust optimization framework to account for delineation uncertainties in prostate HDRBT. van der Meer et al.^24^ addressed organ reconstruction variability by optimizing dwell times over multiple reconstructed anatomy scenarios. Morén et al.^25^ applied scenario based robust optimization to prostate intensity modulated brachytherapy with rotating shields to mitigate rotational uncertainty. More recently, Kennedy et al.^26^ quantified the clinical impact of uncertainties in prostate HDRBT, reporting worst case degradations in $D_{90}$ on the order of 27% under combined uncertainty sources. In a larger cohort study, Kennedy et al.^27^ evaluated robustness across dozens of plans and uncertainty realizations. Building on these findings, Kennedy et al.^28^ developed a probabilistic robust optimization genetic algorithm for prostate HDRBT that optimizes dwell times against large ensembles of uncertainty scenarios. Robust optimization of needle or catheter configurations has also been studied by Gerlach et al. for robotic HDR prostate brachytherapy and by van der Meer et al. using robust evolutionary bi‐objective optimization.^29^, ^30^ Collectively, these methods demonstrate that robust optimization can improve the stability of $D_{90}$ target coverage and OAR dose metrics under uncertainty. However, existing approaches generally separate catheter configuration and dwell time optimization, either through candidate configuration evaluation or sequential optimization frameworks in which catheter placement and dwell time are decoupled. In contrast, the present work formulates a unified robust optimization framework in which catheter configuration selection and dwell time optimization are performed jointly and simultaneously while explicitly accounting for geometric uncertainties during planning.

In this work, an inverse planning framework is proposed to improve robustness against catheter insertion uncertainties in whole‐gland HDRBT prostate treatments while using the clinical implant as the starting geometry. Distinct from prior methods that assume rigid linearity, this study explicitly includes free‐hand, curved catheter geometries to better represent clinical reality. The method is the first to utilize a group minimax concave penalty (gMCP) to jointly optimize catheter positions and dwell times. By coupling this selection mechanism with a surface dose gradient penality (SDGP) at OAR interfaces, the algorithm identifies catheter configurations that are geometrically safe under insertion uncertainty. The framework's ability to maintain clinically acceptable treatment plans with robust PTV coverage and OAR sparing under Monte Carlo simulated insertion errors is evaluated, comparing results to the clinically delivered treatment plans.

## 2 METHODS

### 2.1 General framework

The brachytherapy treatment planning optimization problem was formulated to simultaneously determine optimal catheter positions and dwell times that achieve prescribed dose coverage in target volumes while minimizing dose to OARs. The optimization problem was cast in the canonical form suitable for solution via the Fast Iterative Shrinkage‐Thresholding Algorithm (FISTA):

(1) $$\begin{array}{cc} {\text{minimize}}_{x}\; & f(x)+g(x)\\ \text{subject to}\; & x\geq 0 \end{array}$$

where $x\in R^{n}$ represents the vector of non‐negative dwell times at n dwell positions, f(x) denotes the smooth dose fidelity term that ensures adherence to clinical objectives, and g(x) encompasses potentially non‐smooth regularization terms that promote desirable plan characteristics, such as catheter sparsity.^20^, ^31^

### 2.2 Dose calculation model

The dose distribution was computed according to the TG‐43U1 formalism.^32^, ^33^, ^34^ This was expressed in matrix form as $D=S_{K}\cdot Ax$, where $A\in R^{m\times n}$ represented the dose influence matrix containing dose contributions from unit dwell time at each of the n dwell positions to all m voxels, and $S_{K}$ denoted the reference air kerma strength of the radioactive source. Each element $a_{ij}$ of the dose influence matrix encoded the dose rate contribution from dwell position j to voxel i, accounting for dose rate constant, geometry function, radial dose function, and anisotropy function as specified in the TG‐43U1 protocol. The Flexisource Ir‐192 source model parameters were taken from the GEC‐ESTRO consensus data.^35^

### 2.3 Dose fidelity formulation

The dose fidelity term f(x)=Γ(Ax) quantifies deviations of the calculated dose from the prescribed clinical objectives. It is formulated as a sum of quadratic penalties applied on a voxel‐by‐voxel basis for violations of specified dose limits. The function penalizes underdosing below a minimum threshold ($d_{\min,j}$) and overdosing above a maximum threshold ($d_{\max,j}$) for target volumes (T), while only penalizing doses above a maximum tolerance ($d_{\max,k}$) for organs at risk (O):

(2) $$\begin{array}{cc} \Gamma (Ax)= & \sum_{j\in T}w_{j,\min}{\left\|{\left(d_{\min,j}-A_{j}x\right)}_{+}\right\|}_{2}^{2}\\ & +\sum_{j\in T}w_{j,\max}{\left\|{\left(A_{j}x-d_{\max,j}\right)}_{+}\right\|}_{2}^{2}\\ & +\sum_{k\in O}w_{k,\max}{\left\|{\left(A_{k}x-d_{\max,k}\right)}_{+}\right\|}_{2}^{2} \end{array}$$

Here, $A_{j}x$ and $A_{k}x$ are the dose vectors for voxels in a target structure j and an OAR k, respectively. The positive part function ${\left(z\right)}_{+}=\max\left(0,z\right)$ ensures that penalties are only applied when a dose limit is violated. The weights $w_{j,\min}$, $w_{j,\max}$, and $w_{k,\max}$ allow differential emphasis on target underdose, overdose, and OAR sparing objectives.

### 2.4 Regularization terms

#### 2.4.1 Candidate catheter generation

For optimization‐based catheter selection, a set of candidate catheter positions was generated by expanding the clinical catheter configuration. Starting from the original clinically placed catheter positions, four additional catheter trajectories were created by translating each clinical catheter by ±5 mm in the anterior‐posterior (x) and left‐right (y) directions. This cardinal direction expansion produced five candidate positions for each clinical catheter, the original position plus four shifted variants. Dwell positions falling outside the PTV Loading structure, defined as the prostate PTV expanded by a 3 mm margin with voxels intersecting OARs removed, were cropped, ensuring that all candidate dwell positions remained within clinically relevant regions. The prostate PTV was taken from the clinically approved DICOM structure set used for treatment planning. No additional CTV‐to‐PTV margin was applied. The PTV Loading structure was used only to restrict candidate dwell positions. To assess whether this method resulted in the selection of closely spaced catheters, a catheter‐spacing audit was performed across the 32 patient cohort. Pairwise centerline‐to‐centerline distances were calculated for all selected catheters, and the number of selected‐catheter pairs separated by less than 3 mm was recorded.

#### 2.4.2 Group sparsity via group minimax concave penalty (gMCP)

**FIGURE 1:**
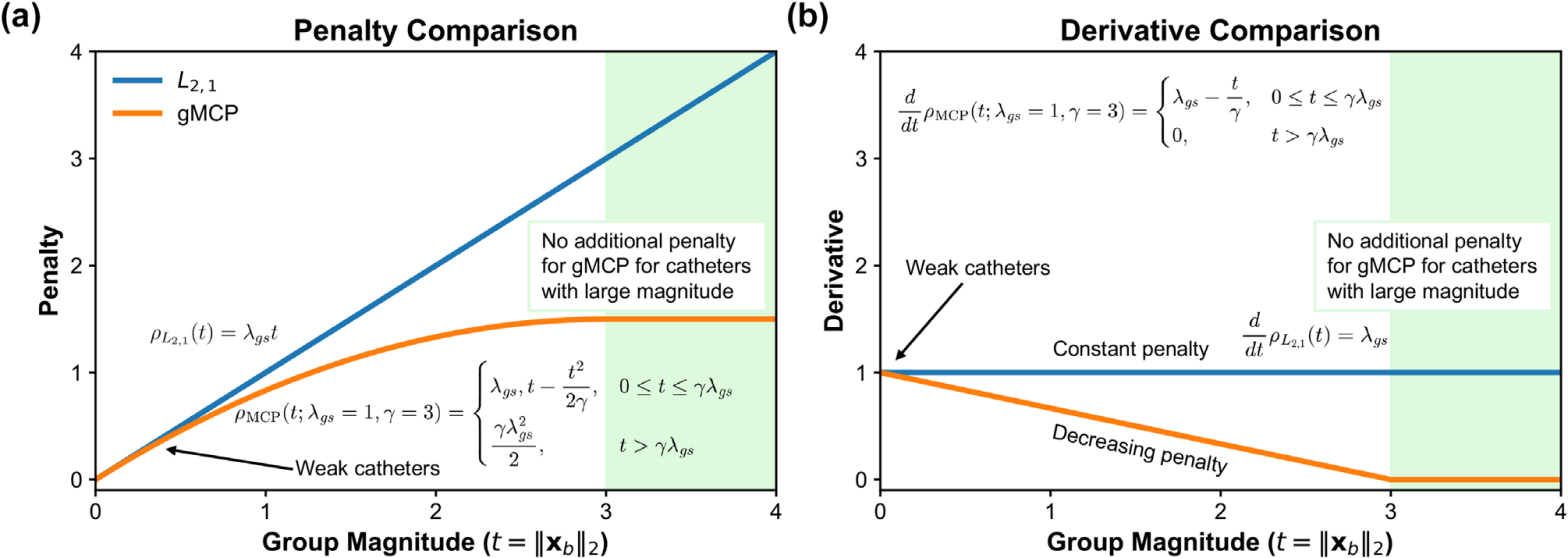
Comparison of Group Minimax Concave Penalty (gMCP) and Group Lasso ($L_{2,1}$) regularization for catheter selection. **(a)** The penalty value plotted against the group magnitude ($t=∥x_{b}{∥}_{2}$) of catheter dwell positions. The convex $L_{2,1}$ penalty (blue) increases linearly, which introduces systematic shrinkage bias by continuously penalizing large coefficients. In contrast, the non‐convex gMCP (orange) transitions to a constant value for large magnitudes (green shaded regions), effectively removing the penalty for strong catheters to ensure unbiased estimation. **(b)** The derivative (gradient) of the penalty functions. While both penalties provide firm thresholding to eliminate weak catheters (small magnitudes), the gMCP derivative vanishes for large magnitudes, avoiding the over‐penalization inherent to the $L_{2,1}$ approach.

Catheter selection was achieved through group sparsity regularization, which encourages entire catheters (groups of dwell positions) to be eliminated rather than individual dwell positions. We employed the minimax concave penalty (MCP), a non‐convex regularizer that provides unbiased estimation for large coefficients while maintaining sparsity‐inducing properties:

(3) $$g_{\text{sparsity}}\left(x\right)=\sum_{b\in B}\rho_{\text{MCP}}(∥x_{b}{∥}_{2};\lambda_{gs},\gamma )$$

where B is the set of all candidate catheters, $x_{b}$ is the vector of dwell times within catheter b, and $\lambda_{gs}$ is the sparsity regularization parameter. The MCP function is defined as:

(4) $$\rho_{\text{MCP}}\left(t;\lambda_{gs},\gamma \right)=\left\{\begin{array}{cc} \lambda_{gs}t-\frac{t^{2}}{2\gamma } & \text{if}\;t\leq \gamma \lambda_{gs}\\ \frac{\gamma \lambda_{gs}^{2}}{2} & \text{if}\;t>\gamma \lambda_{gs} \end{array}\right.$$

where γ=3.0 controls the transition from penalty to no‐penalty regions.^36^ Unlike the convex group $L_{2,1}$ penalty, MCP provides firm thresholding that eliminates weak catheters while leaving strong catheters essentially unpenalized, avoiding systematic shrinkage bias (Figure 1). A similar approach was previously used for EBRT beam angle optimization.^37^ The sparsity regularization strength is controlled by $\lambda_{gs}$ inside $\rho_{\text{MCP}}$.

#### 2.4.3 Surface‐restricted dose gradient penalty (SDGP)

To reduce sensitivity to small catheter‐position errors near critical structures, a surface‐restricted dose‐gradient penalty (SDGP) was added to discourage steep dose changes at OAR boundaries. The SDGP is an implicit robustness regularizer rather than a scenario‐based robust optimization term. It does not sample shifted‐catheter scenarios during optimization. The underlying intuition is that, for a small spatial displacement, the local dose perturbation is approximately proportional to the local spatial rate of dose change in the direction of displacement. Therefore, steep dose variation near an OAR boundary can cause small catheter‐position errors to produce large OAR dose changes.

The penalty is evaluated only on OAR surface voxels. For each OAR k, the surface voxel set $S_{k}$ is obtained from its binary mask using one‐voxel erosion, followed by subtraction of the eroded mask from the original mask. At each optimization iterate, the current summed dose distribution is first calculated from the current dwell‐time vector x. The terms ${\left(\partial_{d}D\right)}_{i}$ denote signed central finite‐difference estimates of the local dose change per unit distance at OAR surface voxel i in direction d∈{x,y,z}. For each direction, the dose at the neighboring voxel one index step in the positive direction is subtracted from the dose at the neighboring voxel one index step in the negative direction, and the result is divided by twice the voxel spacing in that direction. Thus, ${\left(\partial_{d}D\right)}_{i}$ has units of Gy/mm. Because the summed dose distribution is calculated from the current dwell‐time vector, all dwell positions with nonzero dwell time contribute to ${\left(\partial_{d}D\right)}_{i}$, whereas catheters removed by group sparsity have zero dwell time and therefore do not contribute to the SDGP term.

Given a component‐wise gradient threshold T in Gy/mm, the hinge‐squared penalty is defined as

(5) $$\phi \left(u\right)={\left[|u|-T\right]}_{+}^{2},\;{\left[u\right]}_{+}=\max\left(0,u\right).$$

The SDGP regularizer is

(6) $$g_{\mathrm{SDGP}}\left(x\right)=\lambda_{\mathrm{SDGP}}\sum_{k\in O}\sum_{i\in S_{k}}\sum_{d\in \{x,y,z\}}\phi \left({\left(\partial_{d}D\right)}_{i}\right),$$

where O is the set of OARs and $\lambda_{\mathrm{SDGP}}$ controls the regularization strength. In this work, T=0.5Gy/mm was used. A component‐wise penalty was used rather than a penalty on the gradient magnitude so that the threshold T is applied directly to each directional finite‐difference component. This avoids penalizing voxels where multiple subthreshold directional components combine to produce a large gradient magnitude, thereby limiting the penalty to steep directional dose changes at the OAR boundaries. This formulation promotes smoother dose variation at these boundaries without directly constraining gradients within the target that are intrinsic to brachytherapy and needed to maintain coverage.

### 2.5 Complete optimization problem

Combining the dose fidelity and regularization components, the complete optimization problem is formulated as:

(7) $$\begin{array}{cc} {\text{minimize}}_{x}\; & \Gamma \left(Ax\right)+g_{\text{SDGP}}\left(x\right)+g_{\text{sparsity}}\left(x\right)\\ \text{subject to}\; & x\geq 0 \end{array}$$

where Γ(Ax) is the dose fidelity term enforcing clinical objectives, $g_{\text{SDGP}}\left(x\right)$ is the surface‐restricted dose gradient penalty for smooth dose distributions at OAR boundaries, and $g_{\text{sparsity}}\left(x\right)$ is the group MCP penalty for catheter selection.

### 2.6 Solution method

The optimization problem was solved using (FISTA).^31^ The smooth term is defined as

(8) $$f\left(x\right)=\Gamma \left(Ax\right)+g_{\mathrm{SDGP}}\left(x\right),$$

and the non‐smooth term as

(9) $$g\left(x\right)=g_{\text{sparsity}}\left(x\right).$$

FISTA iteratively performs a gradient descent step on f followed by a proximal operator application on g:

(10) $$\begin{array}{c} y_{k}=x_{k}+\frac{k-1}{k+2}\left(x_{k}-x_{k-1}\right) \end{array}$$

(11) $$\begin{array}{c} v=y_{k}-\tau \nabla f\left(y_{k}\right) \end{array}$$

(12) $$\begin{array}{c} x_{k+1}={\text{prox}}_{\tau g}\left(v\right), \end{array}$$

where τ is the step size determined by performing a backtracking line search. Since $\phi \left(u\right)={\left[|u|-T\right]}_{+}^{2}$ is differentiable for T>0, $g_{\mathrm{SDGP}}$ is handled through ∇f. For catheter selection, let b index catheters and let $x_{b}$ denote the vector of dwell times in catheter b (all dwell positions along that catheter). The group MCP proximal operator is applied independently to each catheter block $v_{b}$ of v, where $v_{b}$ contains the entries of v associated with catheter b and $r_{b}={∥v_{b}∥}_{2}$. The resulting closed‐form firm‐thresholding rule is

(13) $$\begin{array}{cc} & {\mathrm{prox}}_{\tau g_{\text{sparsity}}}\left(v_{b}\right)=\\ & \left\{\begin{array}{cc} 0, & r_{b}\leq \tau \lambda_{gs},\\ \frac{1}{1-1/\gamma }\left(1-\frac{\tau \lambda_{gs}}{r_{b}}\right)v_{b}, & \tau \lambda_{gs}<r_{b}\leq \gamma \tau \lambda_{gs},\\ v_{b}, & r_{b}>\gamma \tau \lambda_{gs}, \end{array}\right. \end{array}$$

with γ>1.^36^, ^38^, ^39^ In the limit γ→∞, the transition term approaches the standard group soft‐thresholding form, while γ→1 approaches hard group thresholding.^38^ A standard choice of γ=3.0 was used. This operator has three regimes: a hard‐threshold region that removes weak catheters, a transition region with scaled shrinkage, and a no‐penalty region where strong catheters are unchanged.

For scenarios using generated candidate catheters, the sparsity weight $\lambda_{gs}$ is linearly increased during optimization at a rate of 1% per iteration to achieve a target number of active catheters matching the clinical treatment plan. For scenarios using clinical catheter positions, the group sparsity term is disabled ($g_{\text{sparsity}}=0$) since catheter selection has already been performed clinically. Convergence was determined by monitoring the relative change in the objective function between iterations, with a tolerance of $1\times 10^{-7}$. A dose grid of 2.0 mm voxel resolution was used for optimization, and 1.0 mm voxel resolution was used for dose calculations.

### 2.7 Robustness evaluation methodology

**FIGURE 2:**
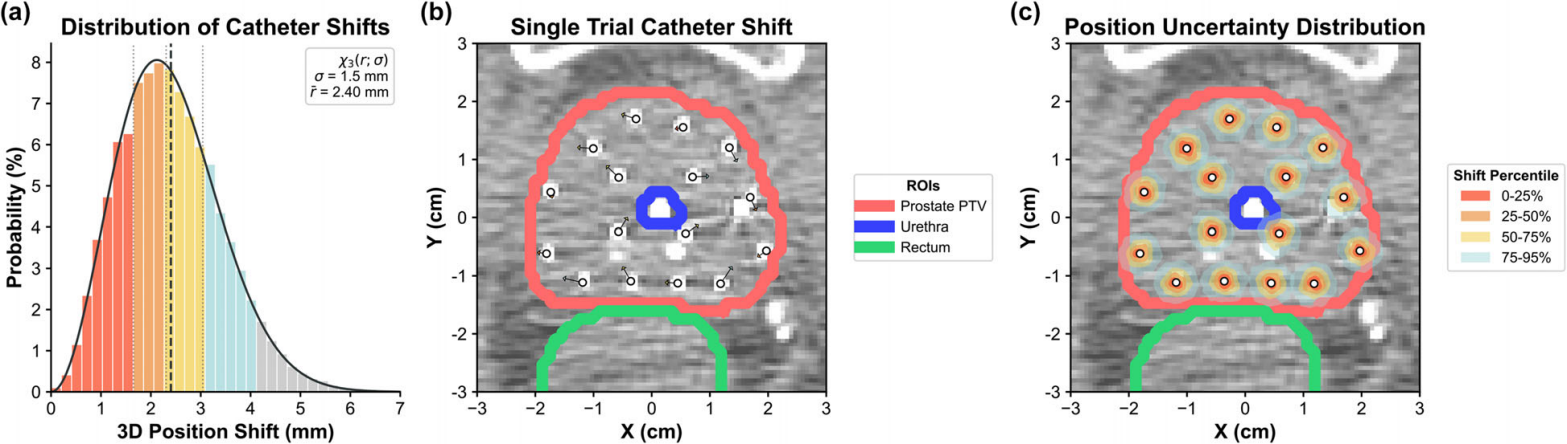
Visualization of the Monte Carlo catheter displacement simulation for the σ=1.5 mm scenario. **(a)** Probability distribution of 3D displacement magnitudes. Independent Gaussian perturbations in each Cartesian axis (x,y,z) produce a chi distribution ($\chi_{3}$) for the total displacement, with mean $\bar{r}=2.40$ mm. Histogram bars are colored by percentile: 0–25^th^ (red), 25–50^th^ (orange), 50–75^th^ (yellow), and 75–95^th^ (blue). Dotted lines indicate the 25^th^, 50^th^, and 75^th^ percentile boundaries. **(b)** Representative single‐trial catheter displacements shown as arrows on an axial CT slice, with arrow color indicating the magnitude percentile of each shift. Anatomical contours show the prostate PTV (red), urethra (blue), and rectum (green). **(c)** Aggregate position uncertainty for all 1000 simulated trials, displayed as concentric probability density contours around each catheter's nominal position. The color‐coded contours correspond to the same percentile zones as panel (a), illustrating the spatial extent of positional uncertainty for each catheter.

Treatment plan robustness was evaluated using a Monte Carlo simulation of catheter placement errors. For each patient and each uncertainty level, n = 1000 random error instances were simulated. Catheter displacements were modeled as independent Gaussian random variables in each cardinal direction (x,y,z). Robustness was evaluated at multiple uncertainty levels with σ∈{0,0.5,1.0,1.5,2.0,2.5,3.0} mm. For each perturbation, dwell times were held fixed at their optimized values. This reflects the clinical workflow in which dwell times are determined during treatment planning without intra‐procedural re‐optimization. The parameter σ represents the standard deviation of the shift in each Cartesian component (x,y,z). As a result, the magnitude of the 3D displacement follows a Maxwell‐Boltzmann distribution with mean displacement of 1.6σ. For example, the σ=1.5 mm scenario corresponds to a 3D displacement of 2.4 mm, with substantial probability mass extending beyond 4 mm. This extensive sampling (1000 trials per patient per uncertainty level) was designed to capture the distribution of possible insertion errors and the resulting variation in plan quality. Figure 2 illustrates the statistical distribution of catheter shifts for the σ=1.5 mm scenario; equivalent distributions for σ=0.5 mm and σ=3.0 mm are shown in Figures S1 and S2.

The chosen uncertainty levels were selected to span a clinically relevant range of reported catheter positioning errors. Whitaker et al. reported a median single‐catheter displacement of 7.5 mm between planning CT and treatment delivery in 48 implants from 25 patients, with 67% of implants showing displacement ≥5 mm.^40^ Kolkman‐Deurloo et al. performed a simulation study of caudal catheter displacements in fractionated prostate HDR and reported clinically relevant reductions in target coverage even for small, millimeter‐scale, catheter shifts, supporting the need for pretreatment catheter position checks and/or robust planning techniques.^11^ Kennedy et al. used 1.5 mm random displacements per axis, per catheter in their probabilistic robustness evaluation.^27^ The uncertainty range σ∈{0,0.5,1.0,1.5,2.0,2.5,3.0} mm considered here spans these prior studies: σ=1.5 mm produces 3D displacements comparable to Kennedy's test conditions, while σ=2.0–3.0 mm produces distributions with substantial probability mass in the 5–7 mm range, consistent with the displacements observed clinically.

### 2.8 Patient population and study design

Thirty‐two prostate cancer patients previously treated with HDRBT at our institution were retrospectively selected for this study. All patients had localized prostate cancer and received a 15 Gy HDRBT boost as part of their treatment regimen following EBRT. The original clinical treatment plans, based on physician placed catheters, were available for comparison. Target and OAR dosimetric endpoints were evaluated according to the NRG‐GU009 protocol. Institutional review board (study #2024‐0218) approval was obtained for this retrospective analysis.

All plans were normalized to achieve PTV V100% = 95% for fair comparison to the nominal plan. This is consistent with the nominal planning objective and prior simultaneous catheter and dwell‐time optimization studies.^19^, ^20^ Using a common normalization level enabled direct comparison of OAR dose and robustness metrics while minimizing confounding differences in baseline target coverage. DVH‐based metrics were tracked across all positional Monte Carlo trials including target coverage (PTV V100%), and OAR sparing (urethra D1cc, bladder V75%, and rectum V75%). For each metric, the mean and standard deviation were computed across trials. Clinical acceptability was defined according to the NRG‐GU009 protocol HDR boost criteria, with PTV V100% ≥90%, urethra D1cc ≤125% of prescription dose, bladder V75% ≤1 cc, and rectum V75% ≤1 cc. The clinical acceptance rate, defined as the percentage of Monte Carlo trials meeting all constraints simultaneously, was calculated for each uncertainty level. Four optimization strategies were compared: 1) a clinical approach using physician catheter placement with only dwell time optimization, 2) clinical catheter placement plus the SDGP term, 3) gMCP for group sparsity catheter selection, and 4) gMCP plus SDGP. All data fidelity, group sparsity, and SDGP hyperparameters remained fixed across all patients and strategies. For catheter selection methods, optimization continued until the number of active catheters matched the clinical plan.

### 2.9 Statistical analysis

Dosimetric comparisons between optimization strategies were performed using paired Wilcoxon signed‐rank tests, with statistical significance set at p<0.05 and adjusted using Bonferroni correction to p<0.017. Differences in robustness metrics, including the mean values and clinical acceptability rates across optimization strategies, were evaluated using the same non‐parametric approach. All statistical analyses were performed using Python (version 3.11) with the SciPy statistical package.^41^

## 3 RESULTS

### 3.1 Patient population

Anatomical and implant characteristics for the 32 patient cases are summarized in Table 1.

**TABLE 1:** Summary statistics for volumes and plan characteristics for n=32 patients.

Characteristic	Mean ± Standard deviation	Range
PTV volume (cc)	45.2±17.8	21.9–102.3
Bladder volume (cc)	23.3±7.1	10.1–35.3
Rectum volume (cc)	14.6±5.3	3.6–23.5
Urethra volume (cc)	1.6±0.5	0.5–3.0
Clinical catheters	16.8±1.9	14.0–22.0
Total dwell positions	227.8±45.0	167.0–347.0
Active dwell positions	135.5±33.6	85.0–243.0

### 3.2 Nominal plan dosimetric performance

**FIGURE 3:**
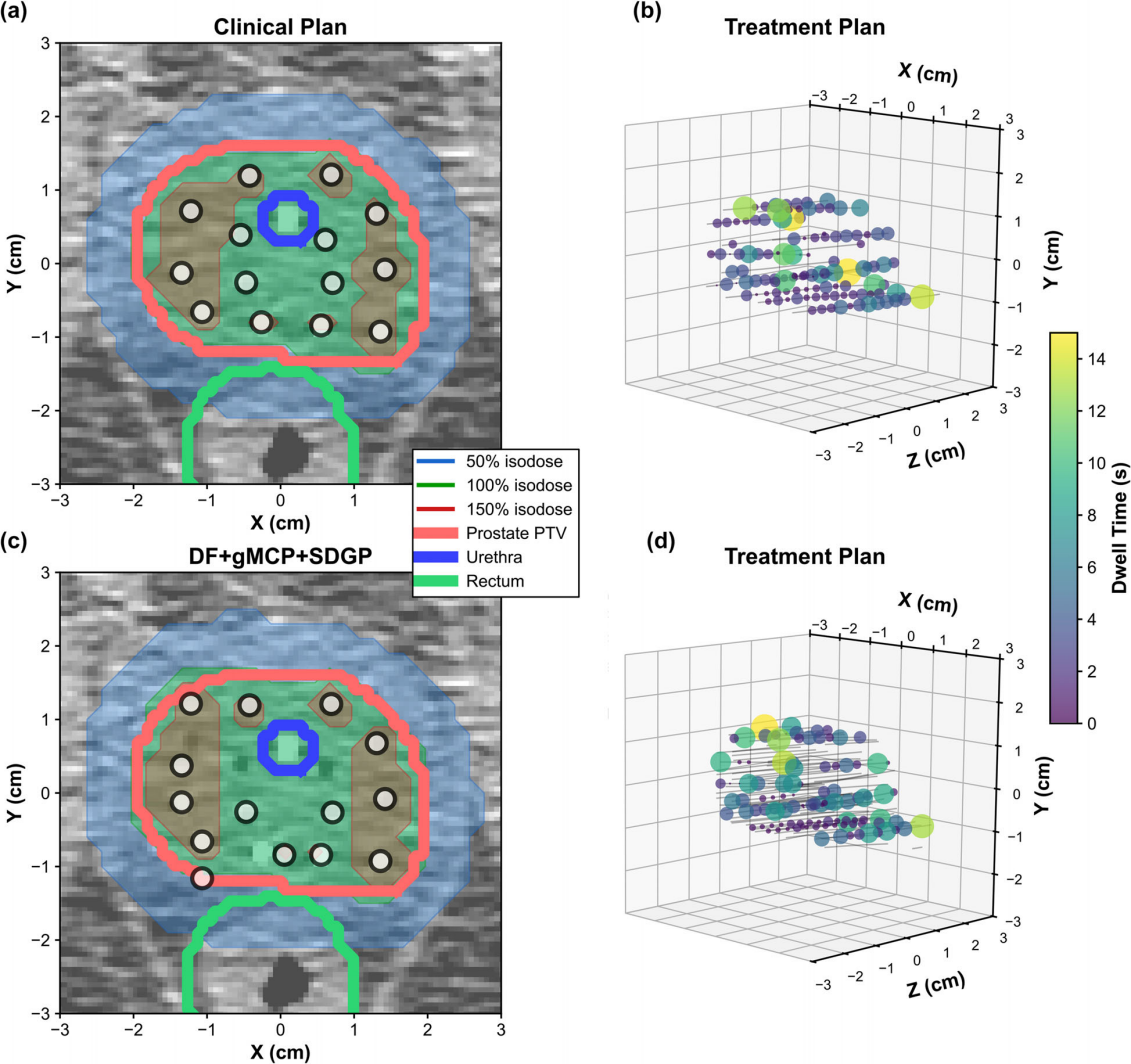
Representative clinical (a,b) and optimized DF+gMCP+SDGP (c,d) plans for an example patient. Panels (a) and (c) show axial dose distributions with 50%, 100%, and 150% isodose lines; prostate PTV (red), urethra (blue), and rectum (green) are overlaid. Panels (b) and (d) show 3D dwell positions, with point color indicating dwell time (s). All plans were normalized to PTV V100% = 95%.

With all plans normalized to PTV V100% = 95%, the optimization algorithms were evaluated for their ability to satisfy OAR constraints. Figure 3 shows a representative dose distribution for an average patient. Table 2 summarizes the nominal dosimetric performance of each optimized plan across all patients. Optimization times per plan were approximately 3 min on a 2023 MacBook Pro with an M3 processor (Apple Inc., Cupertino, CA, USA).

**TABLE 2:** Nominal optimized plan dosimetric comparison.

Parameter	Clinical	DF+SDGP	DF+gMCP	DF+gMCP+SDGP
Target coverage				
PTV V100% (%)^a^	95.0	95.0	95.0	95.0
PTV V150% (%)	25.60±3.44	28.74±4.34 ^*^	25.28±3.16	31.01±4.62 ^*^
PTV V200% (%)	9.86±1.59	10.88±1.78 ^*^	9.89±1.18	11.92±1.72 ^*^
Organs at risk				
Bladder V75% (cc)	0.17±0.22	0.09±0.16 ^*^	0.17±0.25	0.08±0.15 ^*^
Rectum V75% (cc)	0.18±0.22	0.18±0.22	0.20±0.24	0.20±0.24
Urethra $D_{0.1\text{cc}}$ (%)	107.53±4.28	110.39±4.23 ^*^	106.92±3.25	112.57±4.44 ^*^
Urethra $D_{1\text{cc}}$ (%)	84.16±17.55	84.70±17.43	84.02±17.75	85.84±17.35 ^*^

^a^ All plans normalized to nominal PTV V100% = 95%.

^*^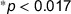 compared to Clinical plan.

### 3.3 Robustness to catheter insertion uncertainties

**FIGURE 4:**
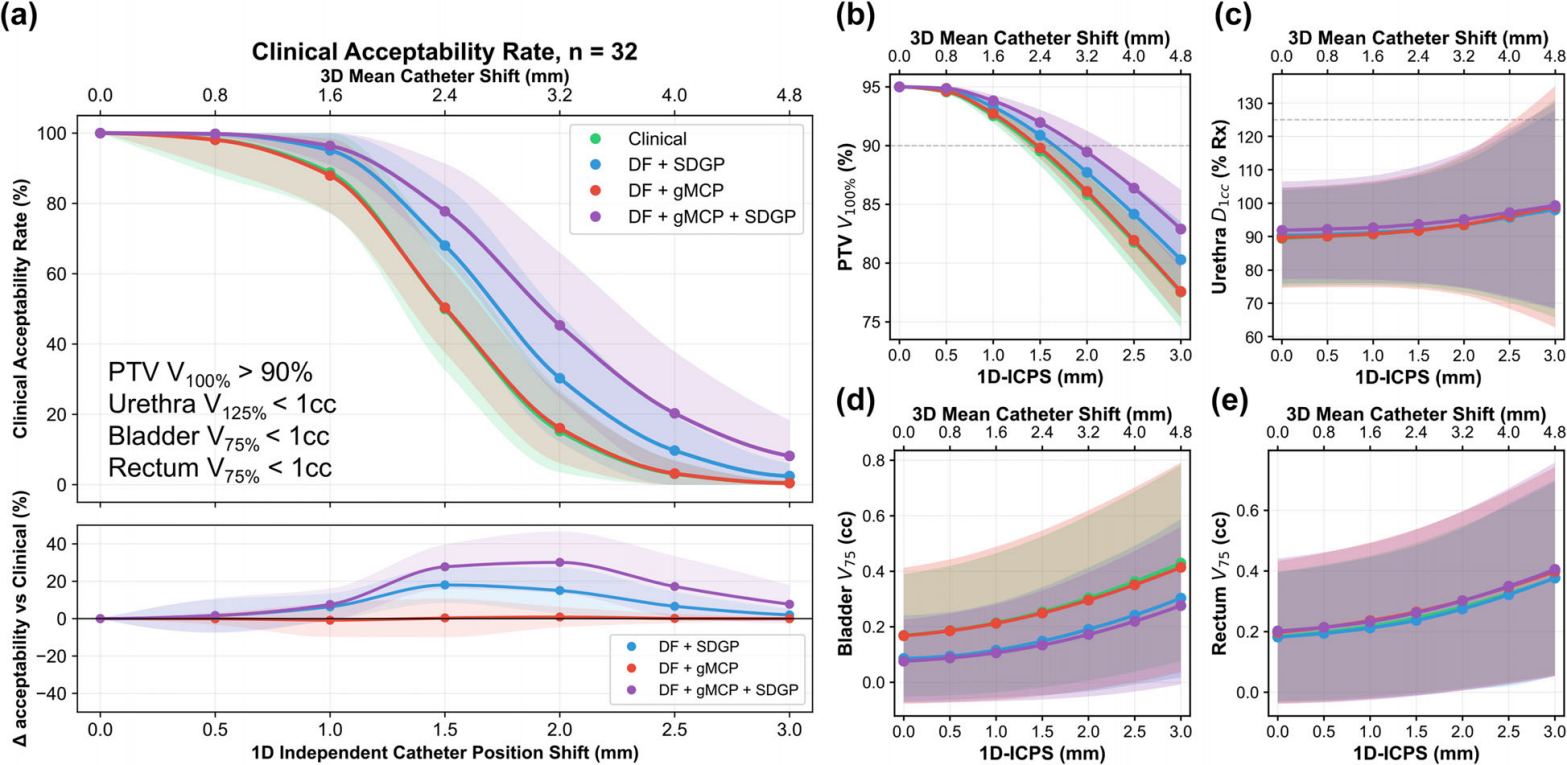
Robustness analysis comparing optimization strategies under increasing catheter position uncertainty (0–3 mm). (a) Clinical acceptability rate (percentage of trials meeting NRG‐GU009 criteria) versus 1D independent catheter position shift, with the corresponding 3D mean shift on the top axis. Shaded bands represent ±1 standard deviation of the patient‐level values about the mean for each catheter shift magnitude. Lines and markers show the mean across n=32 patients. (b–e) Mean DVH metrics with ±1 standard deviation for PTV $V_{100\%}$, urethra $D_{1\text{cc}}$, bladder $V_{75\%}$, and rectum $V_{75\%}$, respectively. Each data point represents 1000 Monte Carlo trials with independent catheter perturbations. Strategies shown: Clinical, DF+SDGP, DF+gMCP, and DF+gMCP+SDGP.

The key innovations of this work, catheter selection via gMCP, SDGP for robust OAR protection, and joint dwell time optimization, were systematically evaluated using positional Monte Carlo simulation of catheter insertion errors. Figure 4 shows the degradation in plan quality metrics as catheter position uncertainty increases from 0 to 3 mm standard deviation. Enhanced robustness compared to clinical planning approaches is demonstrated by the statistically significant increase in clinical acceptability rate. Table 3 quantifies the improvements achieved by robust optimization for the 1.5 mm scenario.

**TABLE 3:** Plan robustness at 1.5 mm 1D catheter position uncertainty for 1000 Monte Carlo trials for *n* = 32 patients.

Parameter	Clinical	DF+SDGP	DF+gMCP	DF+gMCP+SDGP
Target coverage robustness				
PTV V100% mean (%)	89.5±1.2	90.9±1.2 ^*^	89.8±0.9	92.0±1.1 ^*^
Δ Clinical, median [min, max]	—	+1.1[0.1,3.3]	+0.2[−1.3,2.2]	+2.2[0.7,5.1]
*OAR Dose Robustness*				
Bladder V75% mean (cc)	0.3±0.3	0.1±0.2 ^*^	0.2±0.3	0.1±0.2 ^*^
Δ Clinical, median [min, max]	—	−0.1[−0.5,0.0]	0.0[−0.2,0.4]	−0.1[−0.4,0.0]
Rectum V75% mean (cc)	0.2±0.3	0.2±0.3	0.3±0.3	0.3±0.3
Δ Clinical, median [min, max]	—	0.0[−0.1,0.1]	0.0[−0.1,0.1]	0.0[−0.1,0.2]
Urethra $D_{0.1\text{cc}}$ mean (%)	111.1±4.9	113.4±4.7 ^*^	110.7±4.1	115.5±4.8 ^*^
Δ Clinical, median [min, max]	—	+2.1[−4.9,6.7]	−0.1[−11.8,3.5]	+4.3[−9.1,11.7]
Urethra $D_{1\text{cc}}$ mean (%)	91.8±17.2	92.1±16.5	91.8±17.8	93.7±17.7 ^*^
Δ Clinical, median [min, max]	—	+0.7[−5.0,3.0]	−0.1[−3.1,3.2]	+1.3[−4.9,9.9]
Clinical acceptability pass rate (%)				
PTV V100% ≥90%	54.3	71.5	56.8	83.1
Bladder V75% ≤1 cc	95.9	98.5	94.8	98.4
Rectum V75% ≤1 cc	97.5	97.3	95.8	96.0
Urethra V125% ≤1 cc	100.0	100.0	100.0	100.0
All criteria simultaneously	50.0	68.0^*^	50.4	77.7^*^
Δ Clinical, median [min, max]	—	+14.7[2.2,39.4]	+0.6[−21.2,20.6]	+27.0[10.6,49.2]

*Note*: All plans normalized to nominal PTV V100% = 95%. Δ row reports patient‐paired differences in all‐criteria acceptance rate relative to the Clinical plan in the same units as the corresponding metric. Criterion pass rates are patient‐level mean trial pass rates from the Monte Carlo robustness evaluation at σ=1.5 mm.

^*^ p<0.017 compared to clinical plan.

Clinical plan rescaling to PTV V100% = 96% and 97% improved the clinical plan robustness and reduced, but did not eliminate, the robustness advantage of DF+gMCP+SDGP. At σ=1.5 mm, clinical acceptability was 65.5% at 96% normalization and 74.4% at 97% normalization. DF+gMCP+SDGP at its original 95% normalization achieved 77.7%, outperforming the clinical plan even after rescaling. Acceptability curves comparing clinical plans rescaled to 96% and 97% against all methods at 95% are shown in Figure S3.

The selected catheter spacing audit demonstrated that closely spaced selected catheters were uncommon. Clinical and DF+SDGP plans had no selected‐catheter pairs with centerline distances below 3 mm (minimum observed: 3.07 mm) as they both used the same catheter set. DF+gMCP and DF+gMCP+SDGP had 8 and 14 selected‐catheter pairs with centerline distances below 3 mm, respectively, across the entire 32‐patient cohort (minimum observed: 1.53 mm and 1.63 mm).

## 4 DISCUSSION

A novel deterministic inverse planning framework for HDR prostate brachytherapy was developed to integrate explicit robustness objectives with automated joint catheter selection and dwell time optimization. A key advancement in this methodology is the use of the group Minimax Concave Penalty (gMCP) for catheter selection. This work complements prior work by Frank et al., which demonstrated the benefits of non‐convex group sparsity using the $L_{2,1/2}$ norm to improve upon convex $L_{2,1}$ approaches. However, $L_{q}$ norms (where 0<q≤1) impose a penalty that grows with the magnitude of the coefficient, inevitably introducing systematic shrinkage bias. This often necessitates heuristic re‐weighting or post‐selection re‐optimization to restore the full intensity of selected catheters.^19^, ^20^ In contrast, the gMCP employed in this study utilizes a firm thresholding operator. This penalty effectively eliminates weak catheters while transitioning to a zero‐gradient region for large coefficients.^36^ This property ensures that once a catheter is identified as essential, its dwell times are not artificially suppressed by the regularization term, thereby preserving nominal plan quality without the need for heuristic adjustments. In combination with the data fidelity term, this formulation achieves clinical results that closely match the manually planned treatments, as shown in Table 3.

In nominal optimization, the resulting dose distribution can appear smooth for the planned catheter geometry. However, the dose distribution remains sensitive to positional errors as minor catheter shifts can create local hotspots in OARs or reduce target coverage.^23^, ^24^, ^27^ SDGP introduces a robustness tradeoff, in which baseline OAR doses may increase (Table 2) while sensitivity to geometric uncertainty is reduced (Table 3). In this study, DF+gMCP+SDGP improved clinical plan robustness under simulated perturbations, but increased nominal urethra $D_{0.1\mathrm{cc}}$ compared with Clinical and DF+gMCP plans. As shown in Table 2, SDGP also increased PTV $V_{150\%}$ and $V_{200\%}$. This reflects the nominal robustness tradeoff because SDGP penalizes OAR‐surface gradients rather than dose heterogeneity within the target. In a five‐patient SDGP weight pilot, low $\lambda_{\mathrm{SDGP}}$ values gave limited robustness gain. Higher values improved acceptability but progressively increased urethra dose and PTV hot spots, reducing margins from clinical OAR thresholds. At the highest tested values, robustness gains plateaued while plan quality deteriorated sharply. The selected weight was chosen to achieve meaningful robustness improvement while maintaining adequate distance from clinical constraint thresholds. The gMCP then acts as a computationally efficient filter to enforce sparsity on this robust subset of catheters and ensuring that only clinically essential catheters contribute to the optimized plan.

The results from the cohort of 32 patients demonstrate the clinical benefits of this robust formulation (Table 3). Under a realistic catheter insertion uncertainty of σ=1.5 mm, the standard clinical plans exhibited a significant decrease in target coverage, with the mean PTV $V_{100\%}$ dropping from the nominal 95.0% to 89.5%. Notably, the optimization strategy using gMCP‐based catheter selection alone (DF+gMCP) performed similarly to the clinical plans (89.8%), indicating that sparsity regularization alone does not inherently confer geometric robustness. In contrast, the fully robust strategy (DF+gMCP+SDGP) maintained a significantly higher mean coverage of 92.0% (p<0.05) under identical uncertainty conditions. This stability translated directly to clinical viability, as the robust plans achieved a clinical acceptability rate of 77.7% (meeting NRG‐GU009 criteria) compared to only 50.0% for the clinical plans and 50.4% for the non‐robust optimized plans when under 1.5 mm independent Gaussian shifts.^1^

It is important to distinguish the probabilistic robustness evaluation used here from scenario‐based robust optimization. Large uncertainty ensembles have been used for HDR brachytherapy plan evaluation, including in prior work by Kennedy et al. and Balvert et al.^26^, ^42^ In contrast, scenario‐based robust optimization methods can become computationally expensive when many perturbation scenarios are included directly in the objective function, and they have typically optimized dwell times for a fixed catheter geometry rather than using catheter placement as an additional degree of freedom.^11^, ^23^, ^24^ Under independent Gaussian shift errors, the 3D displacement magnitude follows a Maxwell distribution with mean approximately 1.6σ. Therefore, σ=1.5mm corresponds to a mean 3D displacement of approximately 2.4mm, with nonzero probability of larger excursions.^27^, ^40^ By simulating n=1000 independent catheter perturbations per patient, per uncertainty level, this study evaluates the sensitivity of each final plan to stochastic catheter‐placement uncertainty. This Monte Carlo perturbation evaluation is separate from the optimization process, which did not include perturbation scenarios in the objective function.

A common concern in robust optimization is the potential degradation of nominal plan quality. However, the data indicates that this trade‐off is minimal. The robust plans achieved nominal OAR doses comparable to, and in some cases better than, the clinical plans (Table 2). For instance, the nominal Bladder $V_{75\%}$ for the robust plans was 0.08±0.15 cc, compared to 0.17±0.22 cc for clinical plans. While the Urethra $D_{0.1cc}$ was slightly higher in the robust plans (112.57% vs. 107.53%), it remained well within acceptable limits. This implies that the solution space for HDR prostate brachytherapy is sufficiently large to accommodate both high nominal quality and geometric stability, provided the optimization objective is correctly formulated to locate these stable minima using unbiased regularization like gMCP.^36^

This study has limitations inherent to the retrospective design and simulation methodology. The use of independent Gaussian perturbations effectively models random insertion errors but may not fully capture systematic uncertainties such as prostate deformation or bulk patient movement.^5^ Dose calculations were performed using the water based TG‐43U1 formalism, and future work should evaluate robustness under model‐based dose calculation algorithms as recommended by TG‐186 because heterogeneity and applicator effects can alter local gradients and therefore sensitivity.^43^ Additionally, while the sample size of 32 patients provides statistical significance for the observed metrics, validation in a prospective setting is warranted. The current workflow depends on an existing clinical catheter configuration because candidate catheter positions were generated by translating physician‐placed catheters. As a result, the method functions as a robust refinement tool rather than a fully autonomous catheter placement optimization algorithm. However, the proposed optimization framework is independent of the candidate catheter generation strategy and could be extended to candidate sets derived from alternative approaches, including algorithmically generated catheter positions from pre‐procedure volumetric imaging. Finally, as this is a single institutional data set, validation across institutions and prospective workflows is needed to assess generalizability across implant techniques and imaging guidance, and to determine whether robustness‐guided catheter feedback can be integrated into intraoperative decision‐making. SDGP is not prostate‐specific in formulation because it acts on OAR surface dose gradients. However, application to other treatment sites, uncertainty sources, or isotopes would require recalibration of the gradient threshold, $\lambda_{\mathrm{SDGP}}$, and the perturbation model. Gynecologic interstitial brachytherapy is a planned future application. In addition, future work entails evaluating reducing the number of catheters compared to the clinical count, and their effects on the robustness of the plan quality. Integration with a robotic platform to account for mechanical uncertainties is also of interest.

## 5 CONCLUSION

A robust catheter optimization method for HDR prostate brachytherapy was presented that couples unbiased group MCP catheter selection with a surface‐restricted dose gradient penalty to reduce dosimetric sensitivity to catheter placement uncertainty. In a 32‐patient cohort evaluated with probabilistic Monte Carlo perturbations, the robust strategy improved target coverage and increased population level clinical acceptability compared with both clinical planning and non‐robust catheter selection, while maintaining nominal plan quality. These results demonstrate that robustness‐guided catheter selection and surface gradient control can provide a practical mechanism to reduce the probability of plan failure in HDR prostate brachytherapy under realistic geometric uncertainties.

## CONFLICT OF INTEREST STATEMENT

The authors declare no conflicts of interest.

## Supporting information




Supporting Information 1

## Data Availability

The data are not publicly available due to patient privacy and institutional restrictions.
