# Supplementary material for: Robust optimization of catheter positions and dwell times for HDR prostate brachytherapy
Source: Med Phys. 2026 Aug 18;53(9):e70640. doi: 10.1002/mp.70640 (PMC13487368; doi:10.1002/mp.70640)
Supplement: Supplementary file 1 [file MP-53-0-s001.pdf]

Supplementary Material:
Robust optimization of catheter positions and dwell times for HDR prostate brachytherapy

Joseph B. Schulz[1,2,*], Bryan P. Bednarz[1], John M. Floberg[2], Jordan M. Slagowski[1,2,*]

[1]Department of Medical Physics, University of Wisconsin-Madison, Madison, WI, USA
[2]Department of Radiation Medicine, University of Wisconsin-Madison, Madison, WI, USA

[*]Corresponding authors: joey.schulz@wisc.edu, slagowski@wisc.edu

---

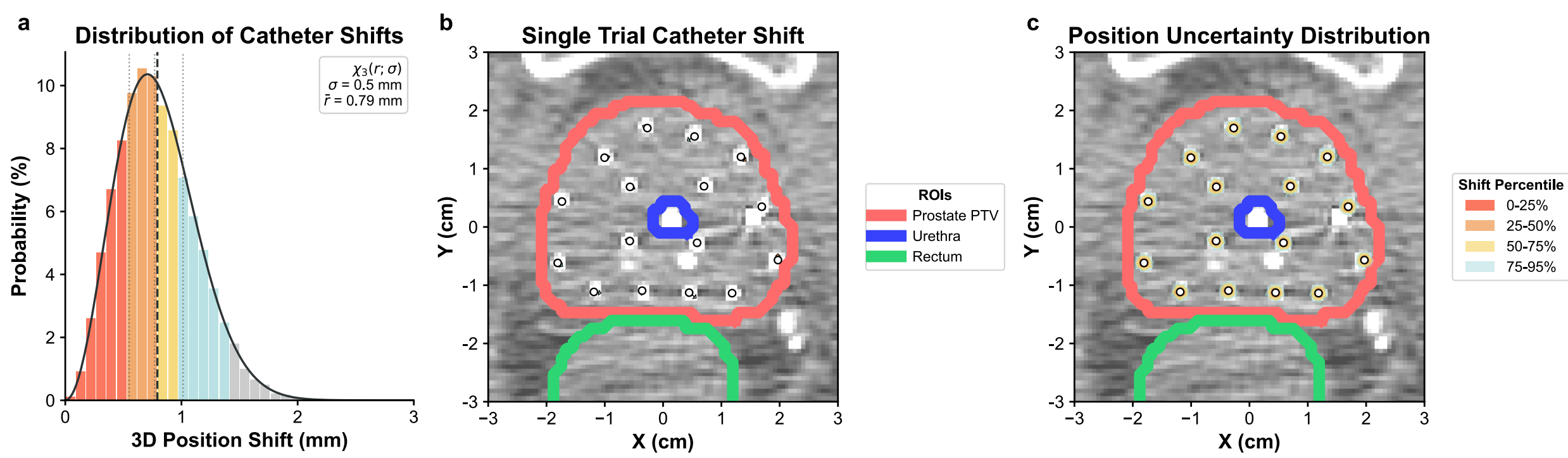

Figure S1: Visualization of the Monte Carlo catheter displacement simulation for the $\sigma = 0.5$ mm scenario. **(a)** Probability distribution of 3D displacement magnitudes. Independent Gaussian perturbations in each Cartesian axis (x, y, z) produce a chi distribution (χ_3) for the total displacement, with mean $\bar{r} = 0.79$ mm. Histogram bars are colored by percentile: 0–25th (red), 25–50th (orange), 50–75th (yellow), and 75–95th (blue). Dotted lines indicate the 25th, 50th, and 75th percentile boundaries. **(b)** Representative single-trial catheter displacements shown as arrows on an axial CT slice, with arrow color indicating the magnitude percentile of each shift. Anatomical contours show the prostate PTV (red), urethra (blue), and rectum (green). **(c)** Aggregate position uncertainty for all 1000 simulated trials, displayed as concentric probability density contours around each catheter's nominal position. The color-coded contours correspond to the same percentile zones as panel (a), illustrating the spatial extent of positional uncertainty for each catheter.

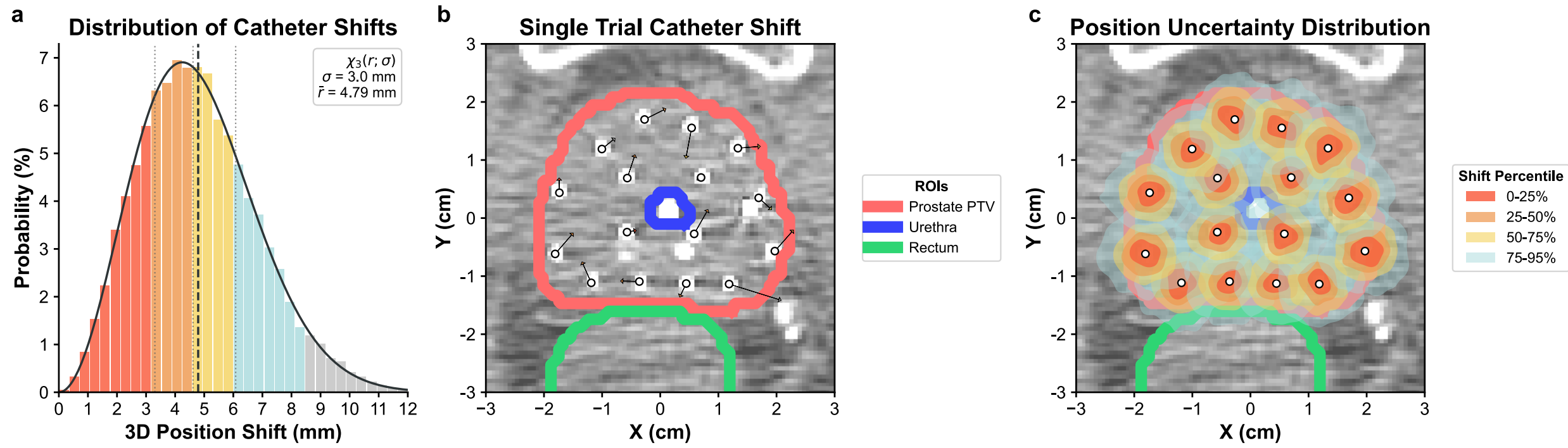

Figure S2: Visualization of the Monte Carlo catheter displacement simulation for the $\sigma = 3.0$ mm scenario. **(a)** Probability distribution of 3D displacement magnitudes. Independent Gaussian perturbations in each Cartesian axis (x, y, z) produce a chi distribution (χ_3) for the total displacement, with mean $\bar{r} = 4.79$ mm. Histogram bars are colored by percentile: 0–25th (red), 25–50th (orange), 50–75th (yellow), and 75–95th (blue). Dotted lines indicate the 25th, 50th, and 75th percentile boundaries. **(b)** Representative single-trial catheter displacements shown as arrows on an axial CT slice, with arrow color indicating the magnitude percentile of each shift. Anatomical contours show the prostate PTV (red), urethra (blue), and rectum (green). **(c)** Aggregate position uncertainty for all 1000 simulated trials, displayed as concentric probability density contours around each catheter's nominal position. The color-coded contours correspond to the same percentile zones as panel (a), illustrating the spatial extent of positional uncertainty for each catheter.

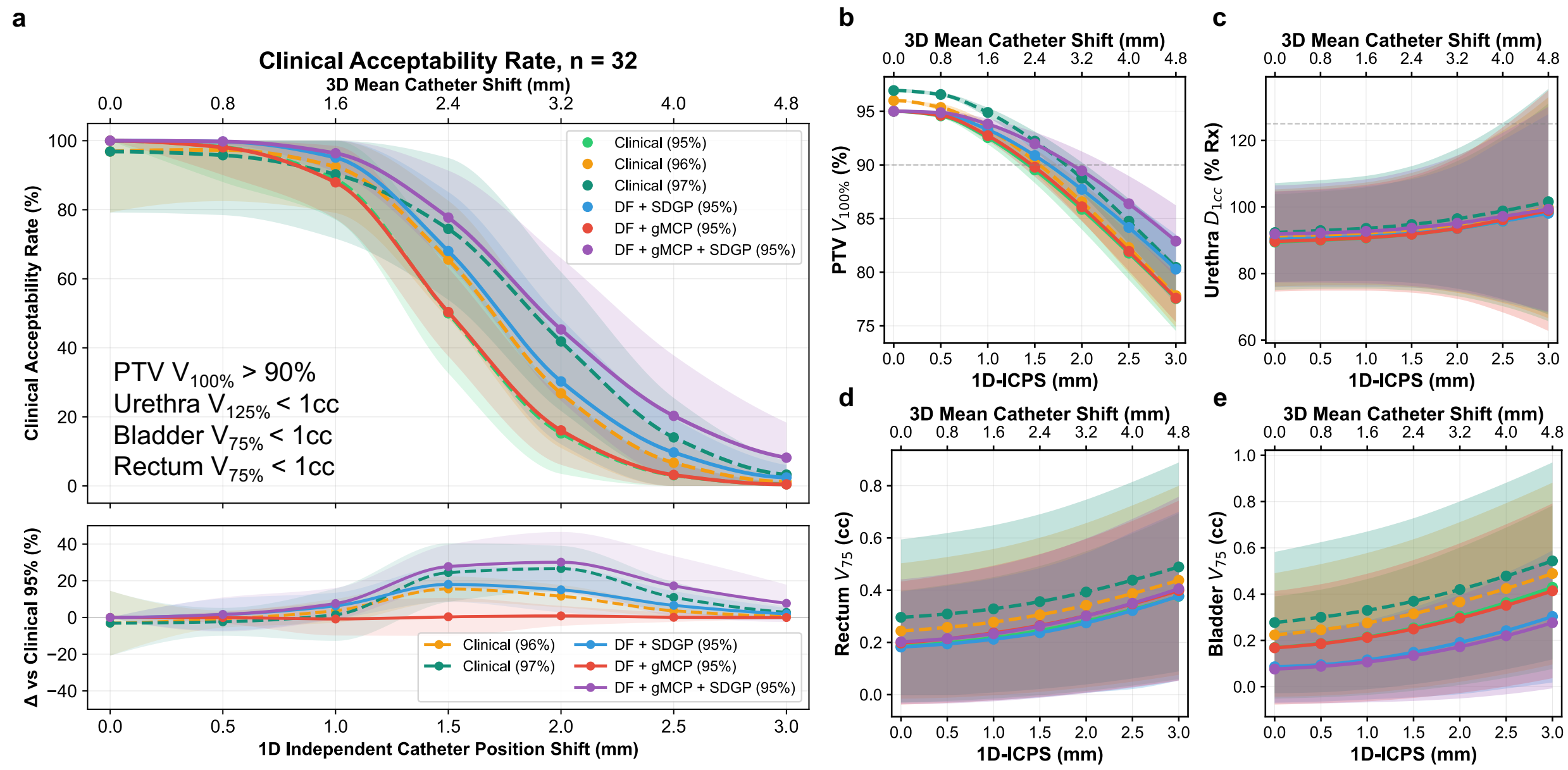

Figure S3: Robustness analysis comparing optimization strategies under increasing catheter position uncertainty (0–3 mm), with clinical plans additionally evaluated at PTV $V_{100\%}$ normalizations of 96% and 97%. **(a)** Clinical acceptability rate (percentage of trials meeting NRG-GU009 criteria) vs 1D independent catheter position shift, with the corresponding 3D mean shift on the top axis. Shaded bands represent ±1 standard deviation of the patient-level values about the mean for each catheter shift magnitude. Lines and markers show the mean across $n = 32$ patients. **(b–e)** Mean DVH metrics with ±1 standard deviation for PTV $V_{100\%}$, urethra D_{1cc}, bladder $V_{75\%}$, and rectum $V_{75\%}$, respectively. Each data point represents 1000 Monte Carlo trials with independent catheter perturbations. Strategies shown: Clinical (95%, 96%, 97%), DF+SDGP, DF+gMCP, and DF+gMCP+SDGP (all at 95%).
